# Systematic Molecularity-Dependent Entropy Errors in Continuum/RRHO Solution Thermochemistry: Origin and Correction

**DOI:** 10.1021/acs.jctc.6c00575

**Published:** 2026-06-30

**Authors:** Aida Rebollar-Zepeda, Mirzam Carreon-Gonzalez, Leonardo Muñoz-Rugeles, Juan Raúl Alvarez-Idaboy

**Affiliations:** † Departamento de Física y Química Teórica, Facultad de Química, 7180 Universidad Nacional Autónoma de México, México City 04510, Mexico; ‡ Laboratorio de Espectroscopía Atómica y Molecular (LEAM), 28014 Universidad Industrial de Santander, Bucaramanga 680002, Colombia

## Abstract

Continuum solvation models often reproduce standard solvation free energies accurately, but this does not ensure that solution-phase reaction free energies are thermodynamically consistent. In common workflows, continuum solvation terms are combined with gas-phase RRHO thermochemical corrections. For molecularity-changing processes such as association, clustering, and transition-state formation, the ideal-gas translational and rotational entropy terms do not cancel and can impose an artificial penalty against associated species. Here, we analyze this effect by decomposing solution-phase association free energies into electronic, differential solvation, and RRHO contributions. Carbon tetrachloride self-association in liquid CCl_4_ provides a simple diagnostic case: the electronic association is favorable, and the differential continuum solvation term is small, yet the final 1 M association free energy remains positive and essentially gas-phase-like. Water and chloroform clusters further show how this molecularity-dependent RRHO penalty accumulates with increasing association. Confinement-based corrections, including Martin–Pratt density scaling and Benson’s free-volume formulation, reduce this artificial destabilization without modifying electronic energies or continuum solvation terms. The main limitation identified here is therefore not the absolute continuum solvation free energy of isolated species but the use of gas-phase RRHO thermochemical corrections to construct reaction free energies in solution. This distinction provides a physical basis for applying condensed-phase translational entropy corrections in association and activation thermochemistry.

## Graphical abstract

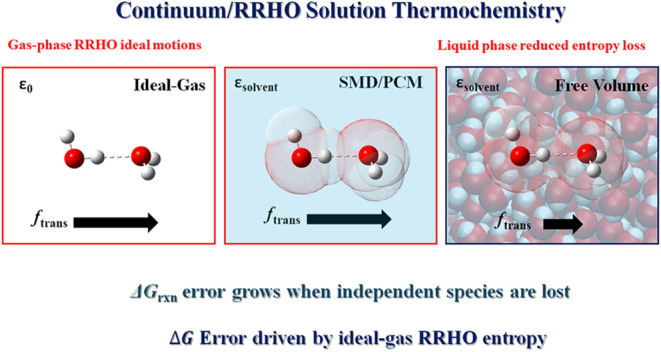

## Introduction

Understanding solvation free energies is essential for describing molecular stability, reactivity, and recognition in condensed phases. Continuum-solvent models—including the Solvation Model based on Density (SMD)^1^ and COSMO^2^ approaches—have achieved notable success in reproducing experimental Δ*G*°_solv_ values across broad classes of chemical systems. Yet, a thermodynamic inconsistency can arise when continuum solvation free energies are combined with ideal-gas RRHO thermochemical corrections to construct solution-phase reaction free energies. Although the electronic component of solvation implicitly includes both enthalpic and entropic contributions from the solvent environment, the molecular entropy is evaluated using the ideal-gas rigid-rotor harmonic-oscillator (RRHO) approximation. In models such as SMD, the solvation term is a parametrized standard transfer free energy composed of continuum electrostatic polarization and empirical short-range contributions, rather than a quantity derived explicitly from molecular translational, rotational, and vibrational partition functions. By contrast, practical reaction free energies in solution are commonly assembled by combining such continuum solvation free energies with gas-phase RRHO thermochemical corrections.^3^

This issue is general and does not depend on the particular continuum model employed. It is closely related to the assumption, often implicit in continuum solvation thermochemistry, that molecular partition-function contributions largely cancel between the gas-phase and solution-phase legs of a thermodynamic cycle.^4,7,15^ Such cancellation may be reasonable for some intramolecular vibrational contributions, but it is much less secure for translational and rotational motions.^4,7,15^ Whether the solvation contribution is obtained from SMD, PCM, COSMO, or related approaches, the usual computational workflow is essentially the same: an electronic energy is combined with a continuum solvation free energy, and gas-phase RRHO thermochemical corrections are then added to obtain a molecular Gibbs free energy. The continuum model may differ in how it evaluates the solvation contribution, but the translational and rotational entropy terms are still taken from the ideal-gas partition functions used in the frequency calculation. Therefore, changing from one continuum model to another does not solve the problem. The inconsistency arises from importing ideal-gas thermochemical corrections into solution-phase reaction free energies, especially when the number of independently moving species changes.

In solution, molecules do not possess the translational freedom assumed in the gas phase. As a result, the RRHO treatment systematically overestimates translational entropy, leading to exaggerated −*T*Δ*S*° penalties whenever the number of independently translating species decreases (Δ*n* ≠ 0; the confinement contribution is intrinsically positive per species, and its effect on Δ*G* depends on the sign of Δ*n*). This mismatch between gas-phase statistical thermodynamics and condensed-phase molecular confinement has been explicitly identified as a major source of systematic error in continuum-solvent free-energy calculations and remains an active subject of methodological refinement in computational chemistry.^4,7,15^ The key issue, however, is not merely the existence of translational-entropy errors, which have been previously recognized, but their magnitude, universality, and systematic propagation across all association processes. In particular, we show that the relevant error becomes apparent in molecularity-changing processes, where ideal-gas translational and rotational entropy terms are combined with continuum solvation free energies. As will be demonstrated below, the critical contribution is not generated by the continuum solvation free energy itself but by the thermochemical workflow used to combine continuum transfer terms with gas-phase partition-function corrections.

This conceptual conflict becomes relevant in association processes such as *A* + *B* → *TS*, addition reactions, and cluster formation although the same thermodynamic principle applies symmetrically to dissociation processes. The problem is not diagnosed primarily from isolated solvation free energies but from reaction free energies in which the number of independently moving species changes. In an *A* + *B* → *C* association or an *A* + *B* → *TS* activation process, the gas-phase RRHO treatment assigns ideal-gas translational and rotational freedom to two independently moving reactants and to one associated product or transition-state structure. Upon subtraction, this mismatch appears as an exaggerated −*T*Δ*S*° penalty against association. In a liquid, much of translational entropy has already been lost upon solvation; therefore, the entropy change associated with forming an encounter complex or a transition state is modest. When ideal-gas entropies are used instead, Δ*S*° becomes excessively negative and −*T*Δ*S*° becomes disproportionately large. The immediate consequence is a systematic and artificial destabilization of any associated species—dimers, clusters, and transition states—relative to separated monomers. This phenomenon has been explicitly documented in continuum solvation studies.^4^ Benchmark studies of association thermodynamics in solution have shown that entropy-related modeling artifacts can shift computed binding free energies by several kilocalories per mole, thereby influencing the mechanistic interpretation of noncovalent assembly.^5,6^

This conceptual conflict is not restricted to weakly bound clusters. It is equally relevant to encounter-complex formation, transition-state formation, and covalent addition reactions, including Diels–Alder-type processes. In such cases, the product or transition state is an ordinary chemical species, and there is no a priori reason to expect its absolute continuum solvation error to become several kilocalories per mole larger than the corresponding errors for the separated reactants. When a large error appears in the computed Δ*G* of reaction or activation, it should not be attributed automatically to a catastrophic failure of the electronic solvation energy of the associated product or transition state. Rather, the relevant diagnostic is the consistency of the complete continuum/RRHO thermochemical cycle used to construct Δ*G* in solution.

Previous studies have quantified this effect with increasing clarity. Ardura, López, and Sordo demonstrated that continuum-based activation free energies for bimolecular reactions are strongly affected by the treatment of the loss of translational degrees of freedom in solution.^7^ Benson’s free-volume formulation offers a physically motivated correction by introducing a cell-like model for molecular confinement in condensed media.^4^ Related theoretical advances by Pratt and LaViolette established quasi-chemical frameworks that account for the restricted motional freedom within liquids,^8^ while Martin, Hay, and Pratt showed that the use of real molecular number densities introduces a finite-volume contribution absent from the conventional ideal-gas reference,^9^ comparable to the one recovered by the Benson approach.^4^ These approaches differ in formalism, but they converge on a common physical point: molecular association in liquids cannot be represented simply as the loss of ideal-gas translational freedom. Modern statistical-mechanical analyses of solvation thermodynamics likewise emphasize that finite-volume confinement constitutes an intrinsic contribution to solvation behavior, as formalized in rigorous thermodynamic treatments of solvent reorganizational energy and entropy.

A fundamental conceptual advance was provided by Garza’s εα formulation,^10^ which bypasses ideal-gas entropy by obtaining solvation entropy directly from the temperature derivative of Δ*G*°_solv_.^10^ Because εα incorporates dielectric response, thermal expansion, and cavity volume changes, it offers a fully thermodynamic way to estimate solvation entropies and enthalpies within a continuum framework. In this sense, εα does not correct the entropy obtained from ideal-gas expressions but replaces it entirely with a thermodynamic quantity derived under condensed-phase conditions. Its relevance here is that it illustrates how solution-phase entropy can be formulated without importing ideal-gas molecular partition functions into condensed-phase thermochemistry. A continuum solvation free energy may reproduce an experimental transfer free energy at a given temperature without providing a unique or explicit decomposition into translational, rotational, vibrational, and solvent-reorganization entropy components. This distinction is directly relevant to reaction thermochemistry, where such components re-enter through RRHO corrections when molecular free energies are assembled. This temperature-derivative perspective aligns with broader efforts to obtain condensed-phase entropies from thermodynamic response functions rather than from gas-phase partition functions, thereby avoiding conceptual inconsistencies inherent to RRHO treatments in liquids.^10^

Related multiscale implicit-solvation approaches have also sought to predict not only solvation free energies but also solvation entropies and heat capacities by combining continuum electrostatics with physically separated nonpolar and specific interaction contributions, such as cavity formation, dispersion, hydrogen bonding, and combinatorial mixing.^11^

Condensed-phase entropy also contains contributions from dynamical solute–solvent correlations, which are absent in static dielectric models. Chandler showed that such correlations define the driving force of hydrophobic assembly,^12^ while Laage and Hynes demonstrated that hindered large-angle jumps mediate water reorientation—an intrinsically non-gas-phase form of molecular motion.^13^ These effects further reduce translational and rotational entropy, reinforcing the conclusion that ideal-gas entropies cannot represent the behavior of molecules in liquids. Condensed-phase entropy is known to emerge from collective solvent structuring rather than from ideal-gas-like molecular motion.

Recent analyses based on explicit evaluation of water oxygen–hydrogen correlation functions have shown that solvation entropies reflect subtle reorganizations of the hydrogen-bond network surrounding the solute, rather than simple translational freedom loss. In such descriptions, the entropic contribution arises from modifications in solvent–solute correlations and local density fluctuations, highlighting that solution-phase entropy is inherently a many-body property of the liquid environment. These microscopic perspectives further reinforce the present argument: the translational entropy obtained from ideal-gas partition functions cannot be interpreted as the physically relevant entropy change accompanying association or solvation in condensed phases.^12–14^

Because continuum models are parametrized to reproduce Δ*G*°_solv_ but not its enthalpic or entropic components, they can match experimental solvation free energies at 298 K while still assigning unphysical entropies. Therefore, accurate monomeric solvation free energies do not guarantee accurate solution-phase reaction free energies when the latter are assembled using gas-phase RRHO thermochemical corrections. Thus, the central issue is not whether a given continuum model provides an intrinsically poor solvation energy for one species, but whether the assembled thermochemical cycle remains internally consistent when ideal-gas translational and rotational terms are carried into solution-phase Δ*G* values.

Recently, alternative approaches based on free-volume or real-gas descriptions within the QM/PCM framework have been proposed to address the overestimation of liquid-phase entropy.^15^ In these models, the translational partition function is modified by replacing the ideal-gas volume with an effective free volume derived from the molecular size and liquid density. While such approaches retain the statistical-mechanical structure of translational partition functions, they recognize that the ideal-gas volume is not an appropriate representation of molecular motion in solution. In the present work, we build on this idea but shift the focus from monomer solvation to molecular-changing reaction free energies. The inconsistency addressed here is therefore not located in the standard continuum solvation free energy alone; it emerges when that transfer free energy is combined with gas-phase RRHO partition-function corrections to construct reaction and activation free energies in solution.

The choice of suitable benchmark systems for this problem is inherently difficult. Ideally, one would require experimentally well-characterized molecularity-changing processes in both the gas phase and solution, involving a single well-defined product or transition state, with negligible conformational ambiguity and minimal solvent-specific interactions. Such systems are rare. In most association or activation processes, the magnitude of the entropy artifact can be comparable to the uncertainty introduced by the electronic-structure method itself. For example, changing the density functional may alter a weak association energy by several kilocalories per mole, and missing a lower-energy conformation of the product or transition state may introduce an error of similar magnitude or even larger if several nearly degenerate structures contribute. These uncertainties can easily obscure the specific contribution that we seek to isolate: the molecularity-dependent penalty introduced by ideal-gas RRHO thermochemical corrections.

The present study builds on this foundation to provide a rigorous and semiquantitative assessment of molecularity-dependent entropy corrections in solution, using water and chloroform clusters as diagnostic systems and bimolecular association as the underlying thermodynamic motif. This issue is particularly relevant for the Quantum Mechanics-based test for the Overall free Radical Scavenging Activity (QM-ORSA) protocol^16^ because the protocol relies on transition-state theory to estimate rate constants for radical–molecule reactions in solution. In such processes, the activation free energy contains a substantial entropic contribution associated with bringing two independently solvated reactants into a single transition-state structure. If the gas-phase RRHO translational and rotational terms are used without an appropriate solution-phase correction, this association penalty can be artificially exaggerated, leading to activation free energies that are too high and rate constants that may be underestimated by several orders of magnitude.

Therefore, the significance of Benson’s correction in QM-ORSA is not merely formal. It directly affects the balance between enthalpic and entropic contributions to ΔG^*‡*^ and, consequently, the quantitative prediction of kinetic behavior in solution. A physically justified entropy correction is essential for preserving the internal consistency of the continuum/RRHO workflow when independently solvated reactants are converted into a single associated species, either a molecular cluster or a transition state. The present analysis clarifies how gas-phase RRHO translational and rotational terms propagate into continuum-based solution free energies, evaluates the consistency of Benson and Martin–Pratt treatments, and examines the implications for association thermodynamics and kinetics. Because Benson’s correction forms a key component of QM-ORSA, particular attention is devoted to assessing its validity and defining the range of conditions under which it remains physically justified. The central question is not whether continuum models can reproduce absolute solvation free energies, but whether the complete continuum/RRHO workflow remains thermodynamically consistent for molecularity-changing processes in solution.

## Computational Methodology

All density functional theory calculations were performed using the meta-hybrid functional M06–2X,^17^ and the 6–311++G­(d,p) basis set was used throughout, with the standard Pople split-valence, polarization, and diffuse-function definitions^18–20^ selected for its reliable performance in noncovalent interactions and thermochemical applications. Geometry optimizations and harmonic vibrational frequency analyses were carried out within the SMD continuum solvation model of Marenich, Cramer, and Truhlar^1^ continuum solvation model, ensuring that all stationary points correspond to true minima (no imaginary frequencies). Thermal corrections to enthalpy and Gibbs free energy were evaluated at 298.15 K within the rigid-rotor harmonic-oscillator (RRHO) approximation as implemented in the quantum-chemical software Gaussian 16.^21^

Solution-phase reaction free energies were reported in the 1 M standard state. Harmonic frequency calculations in Gaussian provide ideal-gas RRHO thermochemical corrections in the 1 atm standard state. Therefore, for bimolecular association processes, *A* + *B* → *C*, the corresponding 1 atm reaction free energy was converted to the 1 M standard state by applying the standard-state correction

$$\Delta G_{1\;M}^{{}^{\circ}}=\Delta G_{1\;\mathrm{atm}}^{{}^{\circ}}+\Delta \mathrm{nRT}\ln\;\left(C^{{}^{\circ}}\mathrm{RT}/p^{{}^{\circ}}\right)$$

where C° = 1 mol L^–1^, p° = 1 atm, and Δ*n* is the change in the number of independently translating species. For a bimolecular association, A + B → C, Δ*n* = −1, and therefore, the 1 atm → 1 M correction is −*RT* ln­(C°*RT*/p°) = −1.89 kcal mol^–1^ at 298.15 K. This conversion was applied consistently to both gas-phase and solution-phase association free energies. It should not be regarded as an optional confinement correction but as the standard-state conversion required to express molecularity-changing reaction free energies at 1 M. Additional confinement-based treatments, such as Martin–Pratt and Benson corrections, were applied separately and only to the translational entropy term; they do not modify electronic energies, optimized geometries, vibrational frequencies, or continuum solvation energies.

The confinement-based corrections considered here differ in the volume used to define the translational entropy reference. The Martin–Pratt treatment replaces the conventional 1 M standard concentration with the real molecular number density of the neat liquid.^9^ For a process in which the number of independently translating species changes, this introduces a density-dependent contribution proportional to *RT*ln (*C*_liq_/1M) for each species lost or gained relative to the 1 M reference. Benson’s formulation instead estimates the translational entropy from an effective free volume available to a molecule in the liquid.^5^ In this approach, the ideal-gas translational volume is replaced by a condensed-phase free volume, thereby representing the restriction of molecular translation by neighboring molecules. Garza’s εα formulation is conceptually different: it obtains solvation entropy from the temperature dependence of the continuum solvation free energy and macroscopic solvent-response parameters, rather than from molecular ideal-gas partition functions. In the present work, Martin–Pratt and Benson are used as practical translational-confinement corrections, whereas Garza’s approach is used as a thermodynamic reference illustrating how solution entropy can be formulated without invoking ideal-gas molecular partition functions.^10^

Benson’s correction was evaluated as a translational free-volume correction to the molecularity-dependent RRHO term. For an *n*-molecular association process, Benson’s formulation leads to

$$\Delta G_{\mathrm{sol}}^{\mathrm{Benson}}≃\Delta G_{\mathrm{gas}}-\mathrm{RT}\ln\left(\frac{n10^{2n-2}}{e^{n-1}}\right)$$

For a bimolecular association, *n* = 2, and therefore

$$\Delta G_{\mathrm{sol}}^{\mathrm{Benson}}=\Delta G_{\mathrm{gas}}-\mathrm{RT}\ln\left(\frac{200}{e}\right)$$

At 298.15 K, this term is

$$\mathrm{RT}\ln\left(\frac{200}{e}\right)=2.55\;\mathrm{kcal}\;\mathrm{mo}l^{-1}$$

Thus, for a bimolecular association at 298.15 K, Benson’s correction lowers the association free energy by 2.55 kcal mol^–1^. This correction acts only on the translational entropy component; it does not modify the electronic energy, optimized structure, vibrational frequencies, or continuum solvation energy.

The Martin–Pratt treatment was implemented as a density-based translational correction. Instead of using the conventional 1 M standard concentration as the reference for independently translating species, the translational reference was replaced by the real molecular number density of the neat liquid, *C*_liq_. For a bimolecular association process, *A* + *B* → *C*, this gives

$$\Delta G_{\mathrm{MP}}=\Delta G_{1M}-\mathrm{RT}\ln\left(\frac{C_{\mathrm{liq}}}{1M}\right)$$

More generally, for an association of *n* monomers into one aggregate,

$$\Delta G_{\mathrm{MP}}=\Delta G_{1M}-\left(n-1\right)\mathrm{RT}\,1n\left(\frac{C_{\mathrm{liq}}}{1M}\right)$$

The Martin–Pratt correction stabilizes association by replacing the dilute 1 M reference with the actual molecular density of the liquid.

In this sense, Martin–Pratt accounts for the macroscopic density of the liquid, whereas Benson attempts to estimate the microscopic free volume available for translation. Both corrections reduce the ideal-gas molecularity penalty, but Benson generally gives a larger correction because the free volume accessible to molecular translation is smaller than the total molar volume of the liquid.

All reported thermodynamic quantities correspond to those at 298.15 K. Cluster association free energies were evaluated relative to separated monomers within a consistent solvation framework, ensuring that differences among models reflect only the treatment of the translational entropy.

For the cluster calculations, the lowest-energy structures were taken from well-established motifs reported in previous theoretical studies and were reoptimized at the level of theory used here.^22,23^ The present analysis does not attempt an exhaustive conformational ensemble treatment. Instead, the clusters are used as controlled diagnostics of the molecularity-dependent RRHO term. For the water dimer, a single dominant hydrogen-bonded arrangement is relevant, whereas for the trimer and tetramer, the most stable hydrogen-bonded structures are well-established from extensive theoretical studies.^22,23^ Alternative arrangements require changes in the hydrogen-bond topology and are sufficiently distinct that their inclusion would constitute an ensemble treatment rather than a simple orientational degeneracy correction. Configurational entropy may become increasingly important for larger clusters, but it is not expected to account for the large positive free energies obtained with the uncorrected continuum/RRHO treatment for the smallest clusters.

## Results and Discussion

A simple diagnostic case is provided by the dimerization of carbon tetrachloride in its own liquid,

$$2\mathrm{CC}l_{4}\to (\mathrm{CC}l_{4}{)}_{2}$$

This system was chosen because it minimizes several sources of ambiguity that complicate more chemically structured association processes. First, as in any simple oligomerization, the covalent bonds are the same on both sides of the equation; the process involves only the formation of a weak intermolecular contact, not a change in molecular connectivity. Second, carbon tetrachloride is highly symmetric and essentially nonpolar. Although different initial mutual orientations of two CCl_4_ molecules are possible, they do not generate chemically distinct association motifs or alternative hydrogen-bonding topologies. Upon optimization, the relevant arrangements converge to the same minimum or to symmetry-equivalent minima related by permutation of identical chlorine atoms. Therefore, no additional configurational entropy term is required to account for multiple chemically distinct dimer structures. In this system, the only significant sources of uncertainty are the intermolecular interaction energy itself and the thermochemical corrections used to convert electronic energies into Gibbs free energies.

The CCl_4_ dimer is stabilized mainly by dispersion and excluded-volume contacts, with no directional hydrogen bonds, proton transfer character, or partially covalent intermolecular interactions such as those present in water dimers. Moreover, because the solute and solvent are the same molecule, the formation of a weak (CCl_4_)_2_ contact in liquid CCl_4_ should not introduce a large differential solvation penalty relative to two separated solvated CCl_4_ monomers. In this limiting nonpolar case, the solution-phase dimerization free energy would therefore be expected to be close to thermoneutral or slightly favorable rather than strongly positive.

For a molecularity-changing association process, the solution-phase reaction free energy obtained in a standard continuum/RRHO workflow can be decomposed as

$$\Delta G_{\mathrm{sol}}^{{}^{\circ}}=\Delta E_{\mathrm{elec}}+\Delta \Delta G_{\mathrm{solv}}^{{}^{\circ}}+\Delta G_{\mathrm{RRHO},\mathrm{corr}}^{{}^{\circ}}$$

where Δ*E*_elec_ is the electronic association energy, ΔΔ*G*°_solv_ is the differential continuum solvation contribution between products and reactants, and Δ*G*°_RRHO,corr_ is the RRHO thermal correction excluding the electronic energy. Thus, Δ*G*°_RRHO,corr_ contains the molecularity-dependent thermal and entropic terms obtained from the frequency calculation, together with the corresponding standard-state conversion when 1 M free energies are reported.

$$\Delta {G_{\mathrm{RRHO},\mathrm{corr}}}^{◦}=\Delta \left({G_{\mathrm{RRHO}}}^{◦}-E_{\mathrm{elec}}\right)$$

Thus, the same expression may be written without ambiguity as

$$\Delta G_{\mathrm{sol}}^{◦}=\Delta E_{\mathrm{elec}}+\Delta \Delta G_{\mathrm{solv}}^{◦}+\Delta \left(G_{\mathrm{RRHO}}^{◦}-E_{\mathrm{elec}}\right)$$

The individual terms are

$$\Delta E_{\mathrm{elec}}=E_{\mathrm{elec}}\left[{\left({\mathrm{CCl}}_{4}\right)}_{2}\right]-2E_{\mathrm{elec}}\left[{\mathrm{CCl}}_{4}\right]$$

$$\Delta \Delta G_{\mathrm{solv}}^{◦}=\Delta G_{\mathrm{solv}}^{◦}\left[{\left({\mathrm{CCl}}_{4}\right)}_{2}\right]-2\Delta G_{\mathrm{solv}}^{◦}\left[{\mathrm{CCl}}_{4}\right]$$

$$\Delta G_{\mathrm{RRHO},\mathrm{corr}}^{◦}=\left(G_{\mathrm{RRHO}}^{◦}\left[{\left({\mathrm{CCl}}_{4}\right)}_{2}\right]-E_{\mathrm{elec}}\left[{\left({\mathrm{CCl}}_{4}\right)}_{2}\right]\right)-2\left(G_{\mathrm{RRHO}}^{◦}\left[{\mathrm{CCl}}_{4}\right]-E_{\mathrm{elec}}\left[{\mathrm{CCl}}_{4}\right]\right)$$

This definition avoids double counting of the electronic energy, which is already included in Δ*E*_elec_. The RRHO contribution, therefore, contains only the molecularity-dependent thermal and entropic terms obtained from the gas-phase frequency calculation, together with the required conversion from the Gaussian 1 atm RRHO convention to the 1 M standard state for bimolecular association. This decomposition separates the electronic association energy, the differential continuum solvation term, and the molecularity-dependent RRHO contribution.

For CCl_4_ dimerization, the electronic association energy is favorable both in the gas phase and in the continuum solvent. In the gas phase,

$$\Delta {E_{\mathrm{assoc}}}^{\mathrm{gas}}=-4.86\;{\mathrm{kcal mol}}^{-1}$$

whereas in SMD/CCl_4_,

$$\Delta {E_{\mathrm{assoc}}}^{\mathrm{SMD}/{\mathrm{CCl}}_{4}}=-3.93\;{\mathrm{kcal mol}}^{-1}$$

The differential continuum solvation contribution is therefore modest

$$\Delta \Delta {G_{\mathrm{solv}}}^{◦}=+0.93\;{\mathrm{kcal mol}}^{-1}$$

The full decomposition of the association free energy is summarized in Table 1.

**Table 1:** Decomposition of CCl_4_ Self-Association Free Energies at 298.15 K^a^

quantity	gas phase	SMD/CCl_4_
Δ*E*_elec_	–4.86	–3.93
ΔΔ*G*°_solv_	NA	+0.93
Δ*G*°_assoc_, 1 atm	+5.54	+6.75
Δ*G*°_RRHO,corr_, 1 atm	+10.40	+9.75
standard-state correction, 1 atm → 1 M	–1.90	–1.90
Δ*G*°_assoc_, 1 M	+3.64	+4.85
Δ*G*°_RRHO,corr_, 1 M	+8.53	+7.82

a Values are in kcal mol^–1^

Note that Δ*G*°_RRHO,corr_ denotes Δ­(*G*°_RRHO_ – *E*_elec_), not the total molecular Gibbs free energy reported by the frequency calculation. The notation emphasizes that *E*_elec_ is included only once, through Δ*E*_elec_. The standard-state correction is listed separately so that the 1 atm Gaussian RRHO convention and the final 1 M association free energy can be distinguished explicitly.

The 1 atm values correspond to the direct Gaussian RRHO thermochemical convention. The 1 M values include the standard-state conversion defined in the Computational Methodology Section, which amounts to −1.89 kcal mol^–1^ for a bimolecular association at 298.15 K. This conversion was applied consistently to both gas-phase and solution-phase association free energies.

Thus, the continuum model alone does not make the dimer strongly unfavorable. The large positive free energy appears only after the gas-phase RRHO thermochemical correction is added. After applying the required 1 atm → 1 M standard-state conversion, the solution-phase association free energy becomes

$$\Delta G_{\mathrm{assoc}}^{◦}\left(1M,\mathrm{SMD}/\mathrm{CC}l_{4}\right)=+4.85\;\mathrm{kcal}\;\mathrm{mo}l^{-1}$$

This value is not smaller than the corresponding gas-phase value,

$$\Delta G_{\mathrm{assoc}}^{◦}\left(1M,\mathrm{gas}\right)=+3.64\;\mathrm{kcal}\;\mathrm{mo}l^{-1}$$

as would be expected if the liquid environment were properly represented for a self-association process in the neat solvent. Instead, the calculated association free energy in liquid CCl_4_ is 1.21 kcal mol^–1^ more unfavorable than in the gas phase

$$\Delta G_{\mathrm{assoc}}^{◦}\left(1M,\mathrm{SMD}/\mathrm{CC}l_{4}\right)-\Delta G_{\mathrm{assoc}}^{◦}\left(1M,\mathrm{gas}\right)=+1.21\;\mathrm{kcal}\;\mathrm{mo}l^{-1}$$

Thus, within numerical accuracy, the gas-phase and solution-phase association free energies are still close in magnitude, but the small difference is in the wrong direction: the liquid phase is predicted to disfavor dimerization more than the gas phase. This is physically counterintuitive for a weak, dispersion-dominated contact between two CCl_4_ molecules embedded in liquid CCl_4_, where the associated pair should be at least as compatible with the medium as two separated monomers. In other words, the standard continuum/RRHO workflow does not reproduce the condensed-phase character of the neat liquid; it behaves as if the association still occurred between ideal-gas-like monomers. This provides a direct diagnostic of the inconsistency: the differential continuum solvation term changes the association energy by less than 1 kcal mol^–1^, whereas the molecularity-dependent RRHO term contributes +9.75 kcal mol^–1^ at 1 atm Gaussian convention. After conversion to the 1 M standard state, the net thermochemical penalty relative to Δ*E*_elec_ + ΔΔ*G*°_solv_ remains +7.85 kcal mol^–1^, as shown explicitly in Table 1.

This result can be understood from the thermodynamics of dissolving a molecule in its own liquid, which is closely related to condensation under the appropriate standard-state convention. At the boiling point, the gas and liquid phases are in equilibrium and therefore Δ*G*_cond_ = 0. This equality does not imply that condensation is thermodynamically empty; rather, it reflects the compensation between a favorable enthalpic contribution, Δ*H*_cond_ < 0, arising from cohesive intermolecular interactions, and an unfavorable entropic contribution, −*T*Δ*S*_cond_ > 0, associated with the loss of molecular freedom upon entering the liquid. Above the boiling point, vaporization or desolvation is favored, Δ*G*_vap_ < 0, whereas below the boiling point, the liquid is the thermodynamically stable phase, Δ*G*_vap_ > 0 and Δ*G*_cond_ < 0. Thus, under the standard conditions considered here, CCl_4_ is not a gas-like medium but a stable condensed phase, already characterized by restricted translational and orientational motion. A major part of this entropy loss is translational: in the liquid, molecules no longer move as independent ideal-gas particles but are continuously restricted by collisions with neighboring molecules, excluded-volume, local solvent structure, and viscous friction. Rotational and orientational motions are also hindered by the same condensed-phase environment although generally to a lesser extent than translation. Denying this distinction would be equivalent to denying that diffusion and molecular reorientation depend on the viscosity and microscopic structure of the medium. Therefore, when the same ideal-gas RRHO translational and rotational corrections used for gas-phase molecules are imported into solution-phase thermochemistry, the calculation assigns gas-like motional freedom to species that are already confined by the liquid. This inconsistency may remain partially hidden when the number of independently moving species is conserved because similar errors cancel between reactants and products. It becomes exposed in molecularity-changing processes, such as *A* + *B* → *C*, *A* + *B* → *TS*, or cluster formation, where the number of independently translating and rotating species changes.

For this reason, the analysis begins with deliberately simple diagnostic systems, such as CCl_4_ self-association, in which the electronic association term, the differential continuum solvation term, and the RRHO contribution can be separated explicitly. The purpose of this example is not to reproduce the full statistical complexity of the liquid but to test whether the standard continuum/RRHO workflow preserves the expected condensed-phase tendency of a weakly associated species in its own solvent.

Having established this point for the simplest dimerization motif, we now extend the analysis to small molecular clusters. Clusters are not intended here as structural models of the liquid phase itself. Rather, they provide a controlled thermodynamic framework in which the same molecularity-dependent RRHO term is amplified systematically as the number of independently moving monomers decreases. In other words, cluster formation acts as a magnifying glass for the entropy imbalance already exposed by the CCl_4_ self-association.

To evaluate whether this molecularity-dependent penalty can be reduced by replacing the ideal-gas translational reference with a condensed-phase volume scale, we compared the standard continuum/RRHO result with two simple confinement-based treatments. The Martin–Pratt treatment replaces the conventional 1 M reference with the real molecular number density of the liquid, thereby introducing a macroscopic finite-volume correction associated with the concentration of molecules in the condensed phase. The Benson correction goes one step further by estimating an effective free volume available for molecular translation in the liquid, rather than using either the ideal-gas volume or the total molar volume. These corrections do not modify the electronic energy, the optimized structures, or the continuum solvation energy. They act only on the translational entropy term and therefore provide a direct test of whether the positive cluster free energies arise mainly from the ideal-gas RRHO treatment of molecularity.

The configurational entropy was not explicitly included in the cluster analysis. In principle, multiple nuclear arrangements of a given (H_2_O)_*n*_ cluster could contribute to the statistical weight of the aggregate and decrease its free energy. This effect, however, is expected to be limited for the dimer, trimer, and tetramers. The water dimer has a single dominant hydrogen-bonded arrangement, and the most stable trimer and tetramer structures have been well established from extensive theoretical studies. Alternative arrangements require substantial rearrangement of the hydrogen-bond network and are sufficiently higher in energy that their Boltzmann populations are expected to be small. For the hexamer, where configurational effects are more relevant, we included not only the book structure but also the cyclic structure, which has been reported to be the most stable in Gibbs free energy. Thus, configurational degeneracy may affect larger clusters, but it cannot account for the large destabilization predicted by the uncorrected continuum/RRHO treatment for the smallest clusters.

The relative free energies of (H_2_O)_*n*_ clusters (Table 2) provide a particularly stringent test because the extended hydrogen-bond network of water strongly stabilizes aggregates relative to isolated monomers. Any continuum/RRHO treatment that predicts monotonic destabilization as *n* increases must therefore contain a substantial molecularity-dependent thermochemical artifact.

**Table 2:** Relative Free Energies Δ*G*°_rel_ (*n*) (kcal/mol) for (H_2_O)*_n_* Clusters at 298 K

N	SMD	Martin–Pratt	Benson
1	0.00	0.00	0.00
2	5.02	2.83	2.47
3	12.31	7.93	7.21
4	16.49	9.92	8.84
5	23.29	14.53	13.09
6, book	28.35	17.40	15.60
6, cyclic	29.43	18.48	16.68

Using ideal-gas RRHO entropies, raw SMD predicts steadily increasing destabilization as the cluster grows, reaching values that are clearly incompatible with the known structures and thermodynamics of liquid water. The numerical results are shown in Table 2.

Columns are ordered according to increasing physical plausibility, defined here by the extent to which artificial positive association free energies are reduced.

The raw SMD trend is unphysical: water hexamers are known, from both experiment and high-level electronic-structure calculations, to be substantially more stable than six isolated monomers.^23,24^ High-quality water-cluster studies obtain this behavior from explicit cluster structures, accurate electronic binding energies, and careful treatment of vibrational and, when needed, anharmonic contributions. In contrast, the present continuum/RRHO workflow combines independently solvated molecular free energies with gas-phase translational and rotational entropy terms. The difference between these approaches explains why the uncorrected continuum/RRHO treatment gives the wrong size dependence, whereas confinement-based corrections recover a more physically reasonable trend. Benson’s correction removes most, but not all, of the artifact; the residual bias reflects the fact that RRHO still treats the solvated clusters as though each aggregate possessed gas-phase motional freedom.

It may be argued that small water clusters do not fully reproduce the thermodynamic properties of liquid water because an extended hydrogen-bond network stabilizes the liquid phase. In contrast, the dimer contains only one hydrogen bond. This is true, and the clusters are not intended to be microscopic models of the liquid. However, in a continuum calculation, the average stabilization provided by the liquid environment is already included, in an effective and parametrized way, through the solvation free energy. Therefore, as the number of water molecules in the cluster increases, the computed free energy should not increase monotonically without a bound. Even if the smallest clusters cannot fully reproduce the hydrogen-bond network of the bulk liquid, the association free energy should tend toward a finite condensed-phase limit rather than progressive destabilization. The same conclusion is obtained for chloroform, where no extended hydrogen-bond network and no strong permanent dipoles are present. Thus, the monotonic destabilization predicted by the uncorrected continuum/RRHO workflow cannot be attributed simply to the special hydrogen-bonding character of water; it reflects the same molecularity-dependent entropy penalty in chemically different liquids. For an infinitely large cluster, the thermodynamic description must approach that of the condensed phase rather than diverge from it.

Relative free energies for water cluster growth were computed using different standard-state conventions and entropy corrections. Although no direct experimental Δ*G*° values are available for these association processes, water aggregation is expected to be thermodynamically favorable; therefore, a monotonic increase in Δ*G*° with cluster size is qualitatively unphysical, as indicated by the results and Figure 1. The conventional 1 M treatments yield such an increase. The Benson correction substantially mitigates the artifact and provides the best quantitative agreement with the expected thermodynamic behavior. The effective concentration correction (Martin–Pratt) captures the leading-order effect of number-density confinement and yields the correct qualitative trend but lacks the quantitative accuracy of the free-volume description. Together, these results support the need for size-dependent confinement-based entropy corrections in solution-phase association.

**Figure 1:**
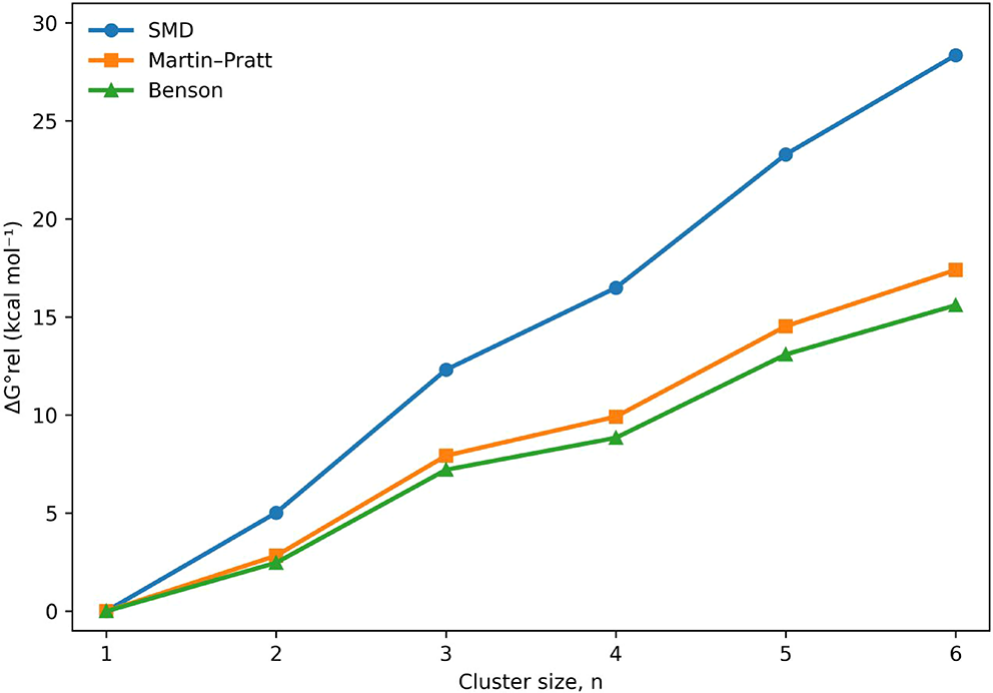
Graphical representation of the relative free energies of (H_2_O)*_n_* cluster association as a function of cluster size using different approaches.

To determine whether this behavior is specific to a strongly hydrogen-bonded liquid, the cluster thermodynamics of chloroform were also analyzed. Stepwise association free energies for (CHCl_3_)*_n_* → (CHCl_3_)_*n*+1_ computed with ideal-gas entropies (1 atm), with the 1 M standard-state correction, and with Benson confinement are summarized in Table 3.

**Table 3:** Stepwise Δ*G*°_add_(kcal/mol) for (CHCl_3_)*_n_* → (CHCl_3_)_*n*+1_ at 298 K

*n*	SMD	Martin–Pratt	Benson
1	0.00	0.00	0.00
2	4.49	3.00	1.94
3	8.75	5.76	3.65
4	14.63	10.15	6.98

In a weakly associating, dispersion-dominated liquid such as CHCl_3_, characterized by weak intermolecular attractions and a low boiling point, aggregate stability arises from cohesive interactions, and the free energy change upon increasing cluster size is expected to be modest, reflecting a balance between dispersion forces and entropic contributions. Instead, gas-phase entropy treatments yield large, positive free energy penalties at each association step, incorrectly suggesting that clustering is strongly disfavored. The confinement corrections significantly reduce these penalties, but a residual RRHO bias persists.

As the cluster size increases, raw gas-phase treatments predict dramatic destabilization relative to the monomer, whereas confinement-corrected approaches produce significantly shallower free-energy profiles. The contrast between raw and corrected values indicates that the apparent instability of chloroform aggregates in standard SMD calculations arises primarily from an entropic artifact rather than from an intrinsic thermodynamic disfavoring of association.

Although chloroform is a weakly associating solvent, the conventional 1 M treatments still yield a steep, nonphysical increase of Δ*G*° with cluster size. The Benson correction substantially reduces this artifact and provides the best quantitative agreement with the expected thermodynamic behavior. The effective concentration correction (“real-concentration CHCl_3_”) also improves the size dependence by capturing the leading-order effect of number-density confinement but remains less accurate than the free-volume description. These results demonstrate that confinement-related entropy effects are essential even in weakly associating solvents.

The comparative analysis of water and chloroform clusters thus provides a clear illustration of how the molecularity-dependent RRHO entropy penalty manifests across liquids with fundamentally different intermolecular interactions. For water, cluster formation is physically stabilized by an extended hydrogen-bond network, yet the uncorrected continuum/RRHO workflow predicts monotonic destabilization as *n* increases. For chloroform, where association is intrinsically weaker and dominated mainly by dispersion forces, the same gas-phase RRHO treatment again yields excessively large positive free energy penalties. In both cases, the dominant source of the artificial destabilization is the same: the use of ideal-gas translational entropy terms in molecularity-changing solution-phase processes.

After application of the Benson correction, the remaining deviation in Δ*G°* for associative processes is small but non-negligible. This residual discrepancy should not be interpreted as purely translational in origin. It may arise from nontranslational entropy contributions, including dynamical solute–solvent correlations, hindered rotational motions, and orientational restrictions that lie beyond a simple free-volume model. It may also reflect limitations in the enthalpic component of the calculation, including the underlying electronic-structure description and the continuum representation of solvent response.^12–14^ For cluster association processes, a complete experimental decomposition into Δ*H°* and −*T*Δ*S°* is generally not available, and the residual error cannot be unambiguously partitioned between enthalpic and entropic terms. Nonetheless, the consistent behavior observed for both water and chloroform indicates that the dominant qualitative artifact is not system-specific but arises from the molecularity-dependent RRHO term in the standard continuum/RRHO workflow.

A comparison of the Martin–Pratt and Benson treatments reveals a clear solvent-dependent trend. For water clusters, the difference between both approaches remains modest and increases gradually with cluster size, whereas for chloroform clusters, the separation is larger. This behavior is physically consistent with the different densities and microscopic free volumes of the two liquids. In highly dense and strongly structured water, the macroscopic density-based correction embodied in the Martin–Pratt approach captures a substantial fraction of the effective translational confinement, leading to results numerically close to Benson’s free-volume model. In less dense chloroform, the same density correction improves the conventional 1 M reference, but does not fully account for the smaller microscopic free volume available for molecular translation.

This distinction is important. The shift from the conventional 1 M standard state to the actual molecular number density of the liquid captures a physically meaningful, density-driven component of translational confinement. At the same time, the remaining difference between Martin–Pratt and Benson indicates that macroscopic density alone is not sufficient for a quantitative description. An additional contribution associated with the microscopic free volume accessible to the solute is required. The stronger stabilization obtained with Benson’s free-volume term therefore reflects a genuine physical distinction between total molar volume and accessible translational free volume, rather than a numerical artifact.

Within this broader picture, Benson’s correction provides a physically interpretable and practically useful reduction of the ideal-gas translational entropy penalty. It represents an approximate reconstruction of the accessible translational phase space in condensed media, rather than an empirical adjustment to the continuum solvation energy itself. Importantly, the correction does not modify electronic energies, optimized structures, vibrational frequencies, or continuum solvation terms; it acts only on the translational entropy component. This makes it compatible with Δ*G°*-based kinetic protocols such as QM-ORSA, where association and transition-state formation are central to the calculation of solution-phase rate constants.^4,16^

Finally, alternative interpretations remain possible within the continuum-solvation community. In particular, some approaches assume that entropy-related effects are partly embedded in the parametrization of continuum solvation models or can be treated through explicit construction of local solvent environments, as in mixed explicit/continuum or cluster–continuum methodologies.^1,3,4,24–26^ The present results do not contradict the success of such parametrized solvation free energies for isolated species. Rather, they show that when continuum solvation terms are combined with gas-phase RRHO thermochemical corrections, molecularity-changing reaction free energies can acquire a systematic ideal-gas entropy penalty. Translational entropy corrections must therefore be considered explicitly when analyzing association, clustering, and activation processes in solution.

## Conclusions

In this work, we present a unified analysis of translational entropy corrections in solution with particular emphasis on their physical interpretation and practical consequences for bimolecular association processes within the standard continuum/RRHO thermochemical workflow. By using CCl_4_ self-association and small-molecule clusters as diagnostic test systems, we isolate the effects of different entropy treatments and systematically assess their impact on molecularity-changing free energies in solution.

Our results demonstrate that solution-phase reaction free energies assembled from continuum solvation terms and gas-phase RRHO thermochemical corrections can systematically overestimate the free energy penalty for association. This behavior arises from the inappropriate application of ideal-gas translational entropy expressions to a condensed-phase environment, where the surrounding solvent intrinsically constrains molecular association. Without correction, this artifact can convert an electronically favorable association into an apparently unfavorable solution-phase process, thereby obscuring the underlying chemistry of association and activation in condensed media.

The CCl_4_ self-association diagnostic illustrates the origin of the problem particularly clearly: the electronic association energy is favorable, and the differential continuum solvation term is small, yet the final 1 M association free energy remains positive and nearly indistinguishable from the gas-phase value. This behavior shows that the dominant penalty does not arise from the continuum solvation term itself but from the molecularity-dependent RRHO contribution introduced when two independently moving reactants are converted into one associated species.

Among the approaches examined, Benson’s translational entropy correction provides a robust and physically transparent improvement, effectively compensating for the missing confinement contribution. Although this approach lacks explicit size dependence, it yields consistent and quantitatively reasonable trends and remains attractive for its simplicity and broad applicability. In contrast, the Martin–Pratt, or real-number-density, treatment introduces a macroscopic finite-volume correction by replacing the conventional 1 M reference with the molecular concentration of the liquid. This correction captures an important part of the confinement effect, whereas Benson’s free-volume treatment provides a more microscopic estimate of the translational volume available in the condensed phase.

Despite their different theoretical formulations, all corrections considered here reflect a common physical principle: molecular association in solution occurs within a finite effective volume that is not captured by ideal-gas standard-state thermodynamics. In this context, effective concentration and free volume approaches can be viewed as complementary approximations to the same underlying confinement effect.

Finally, it is important to recognize that none of the entropy corrections discussed here constitutes an explicit treatment of solvent structure or dynamics. Rather, they provide effective remedies for the thermodynamic inconsistency introduced when continuum solvation free energies are combined with gas-phase RRHO corrections in molecularly-changing reactions.

It is also worth noting that this interpretation is not universally accepted, and alternative views have been advanced within the continuum-solvation community, including treatments in which entropy-related effects are assumed to be partly embedded in parametrized continuum free energies or addressed through mixed explicit/continuum solvation models.^1,3,4,25,26^ Nevertheless, the present analysis highlights the critical role of translational entropy corrections in the reliable interpretation of solution-phase free energies and provides a coherent framework for understanding and comparing alternative approaches. We hope that recognizing the molecularity-dependent nature of these entropy errors will encourage the development of more realistic thermochemical treatments for association and activation processes in solution.

## Data Availability

No data are associated with this article.
